# M2 macrophages participate in ILC2 activation induced by *Helicobacter pylori* infection

**DOI:** 10.1080/19490976.2024.2347025

**Published:** 2024-05-01

**Authors:** Ruyi Peng, Canxia Xu, Linfang Zhang, Xiaoming Liu, Dongzi Peng, Xingcen Chen, Deliang Liu, Rong Li

**Affiliations:** a Department of Gastroenterology, the Second Xiangya Hospital of Central South University, Changsha, Hunan Province, China; b Research Center of Digestive Disease, Central South University, Changsha, Hunan Province, China; c Clinical Research Center of Digestive Diseases of Hunan Province, Changsha, Hunan Province, China; d Department of Gastroenterology, Third Xiangya Hospital of Central South University, Changsha, Hunan Province, China; e Hunan Key Laboratory of Nonresolving Inflammation and Cancer, Changsha, Hunan Province, China; f Department of Gastroenterology, Second Affiliated Hospital of Nanchang University, Nanchang, Jiangxi Province, China

**Keywords:** Group 2 innate lymphoid cells, M2 macrophages, *Helicobacter pylori*

## Abstract

*Helicobacter pylori* (*H. pylori*) causes a diversity of gastric diseases. The host immune response evoked by *H. pylori* infection is complicated and can influence the development and progression of diseases. We have reported that the Group 2 innate lymphocytes (ILC2) were promoted and took part in building type-2 immunity in *H. pylori* infection-related gastric diseases. Therefore, in the present study, we aim to clarify how *H. pylori* infection induces the activation of ILC2. It was found that macrophages were necessary for activating ILC2 in *H. pylori* infection. Mechanistically, *H. pylori* infection up-regulated the expression of indoleamine 2,3-dioxygenase (IDO) in macrophages to induce M2 polarization, and the latter secreted the alarmin cytokine Thymic Stromal Lymphopoietin (TSLP) to arouse ILC2.

## 1. Introduction

*Helicobacter pylori* (*H. pylori*) is a parasitic gram-negative bacillus in the gastric mucosa. Its infection is associated with atrophic gastritis, peptic ulcer, gastric cancer, and gastric mucosa-associated lymphoid tissue lymphoma.^1^ *H. pylori* can modulate the Th1/Th2 and Th17/Treg immune balance, which represent the balance between pro-inflammatory and anti-inflammatory effects, and regulate mucosal immune homeostasis.^2–4^ As for the Th1/Th2 balance, a strong type-1 immunity can be observed in the early phase of *H. pylori* infection, and persistent *H. pylori* infection can promote the secretion of Th2 cytokines, activate the type-2 immunity in the gastric mucosa.^5^ Recently, we reported that in the progression of the *H. pylori* infection associated “inflammatory-cancer” chain (atrophic gastritis, atypical hyperplasia, and gastric cancer), the expression of the Th2 cell-specific transcription factor GATA-3, accompanied by Th2 cytokines IL-4, IL-5, IL-13, were gradually increased in gastric mucosa and peripheral blood of patients infected with *H. pylori*.^6,7^

Th2 cytokines are derived from Th2 cells and the Group 2 innate lymphocytes (ILC2). The ILC2 subset is one of the three major subsets of innate lymphoid cells. ILC2 primarily reside in the mucosal barrier, mesenteric lymph nodes, spleen, and peripheral blood, mediating the mutual promotion between innate immunity and adaptive immunity.^8–10^ Like Th2 cells, ILC2 express GATA-3 and secretes type 2 cytokines in response to IL-25, IL-33, and Thymic Stromal Lymphopoietin (TSLP).^9,11^ In the early stage of infection, ILC2 have been activated while adaptive immunity has not yet been established. ILC2 produces Th2 cytokines, which rapidly promote the establishment of adaptive type-2 immunity. Then, Th2 cells secrete abundant cytokines and act on ILC2 to form a positive interaction loop, which exerts a cascade effect.^12^ Our previous study found for the first time that the ratio of ILC2 cells and Th2 cytokines increased significantly after *H. pylori* infection. To determine whether ILC2 played a role in this process, Th2 cells were cleared from Peripheral Blood Mononuclear Cells (PBMC), and *H. pylori* infection still promoted the secretion of Th2 cytokines.^6^ Accordingly, ILC2 grew and activated in *H. pylori* infection, but the underlying mechanisms remain unraveled.

Macrophages are also one of the essential mediator cells in the early immune response to invading pathogens. They participate in acute and chronic inflammation when encountering *H. pylori*.^13^ M2 macrophages and ILC2 are involved in the mutual promotion, coordination, and development of type-2 immunity.^14–17^ A predominant Th2 and M2-like phenotype was found in patients with gastric cancer.^18,19^ It was tempting to speculate that M2 polarization of macrophages may have something to do with the activation of ILC2 induced by *H. pylori* infection.

This study provides evidence that M2 macrophages participate in ILC2 activation induced by *H. pylori* infection. Moreover, the inner interaction between ILC2 and M2 macrophages was explored, and TSLP derived from macrophages was identified as the essential signal in the process. These findings provide a deeper understanding of the innate immune response in *H. pylori* infection.

## 2. Materials and methods

### 2.1. Bacterial culture, cell culture, and coculture system

The *H. pylori* strains and culture were performed as previously described.^6^ Among them, the *H. pylori* strains of the East Asian type (E), Western type *SS1* (W), and ATCC43504 are CagA^+^ strains. Animal experiments were performed with *H. pylori* strains of E, W, and CagA^−^ type (C). *H. pylori* ATCC43504 was used for all of the in vitro experiments, and the multiplicity of infection (MOI) is 50 if not specified otherwise.

The human gastric epithelial cell line GES-1 and the human acute monocytic leukemia cell line THP-1 were purchased from the Chinese Academy of Sciences Cell Bank. GES-1, THP-1, or freshly isolated human PBMC were grown in RPMI-1640 (Gibco, #11875119) containing 10% fetal bovine serum (Every Green, #13011–8611), 100 U/ml penicillin, and 100 mg/ml streptomycin in a 37°C incubator with 5% CO_2_. Penicillin and streptomycin were withdrawn when cells were cocultured with *H. pylori*.

A transwell chamber (pore size = 0.4 μm) was used for the coculture system. 1 × 10^4^ GES-1 cells were planted overnight on the upper chamber of cell culture inserts, then live *H. pylori* ATCC43504 (MOI = 50) or *H. pylori* lysates (ultrasonic crushing of *H. pylori* ATCC43504) were added into the upper chamber, and 1 × 10^6^ fresh isolated PBMC were separately seeded into the lower culture plates. After coculture for 24 hours, PBMC, GES-1, and supernatants from the lower chamber were collected for further analysis.

### 2.2. PBMC isolation and ILC2 sorting.

The blood samples for PBMC isolation were derived from peripheral blood of healthy volunteers aged 18–75. They had negative results of both *H. pylori* serology and^13^C-urease breath test, with normal routine laboratory results, free of gastrointestinal symptoms, and without past medical history. All specimens were collected with informed consent, and the research program was approved by the Ethics Committee of the Second Xiangya Hospital, Central South University. Heparinized peripheral blood diluted with 1: 1 phosphate-buffered saline (PBS) was layered over an equal volume of Ficoll and centrifuged as per the manufacturer’s instructions. Fluorescent cell sorting was used to separate ILC2, which were defined as Lin^−^GATA-3^+^, Lin^−^CD127^+^CRTH2^+.20,21^ Recombinant human IL-2 (R&D, #BT-002-050), IL-25 (R&D, #1258-IL-025), and IL-33 (R&D, #3625-IL-010) were used to activate ILC2 as a positive control.

### 2.3. Construction and identification of M0, M1, M2 macrophages models

THP-1 was induced by PMA (100 ng/ml, 72 h, Sigma, #16561-29-8) to construct an M0 macrophage model. After successful induction, the M0 surface molecular markers (CD68 and CD11b) were analyzed by flow cytometry (data not shown).

IL-4 (Biolegend, #574006) and IL-13 (Biolegend, #571106) were added to the cell culture medium (10 ng/ml, 48 h). The M1 macrophages (CD11b^+^CD86^+^) and M2 macrophages (CD11b^+^CD206^+^) were detected by flow cytometry. The preliminary results showed that the method we used could induce the formation of more than 90% of M2 macrophages (data not shown).

### 2.4. Animal model

Twenty 4–6 weeks old male C57BL/6 mice were purchased from the Department of Laboratory Animal, Third Xiangya Hospital of Central South University (Changsha, China). Live *H. pylori* were suspended in PBS to 10^9^ CFU/ml. After fasting for 24 h, mice were inoculated with *H. pylori* at a volume of 300 μl by intragastric gavages (once every two days and lasting for a week), respectively. Mice were divided into 5 groups according to different *H. pylori* strains: mice group with no treatment (N), mice group treated with PBS (NC), mice group treated with *H. pylori* strain of East Asian type (E), mice group treated with *H. pylori* strain of Western type *SS1* (W), mice group treated with CagA^−^ *H. pylori* strain (C). All mice were dieted with the same food and sterile water. The mice were sacrificed 6 weeks, 12 weeks, or 24 weeks after intragastric gavages. Rapid urease test (RUT) and pathological biopsy were employed for detecting *H. pylori* infection. Splenocytes were obtained for the preparation of lymphocyte suspensions. Medical ethics protocol was approved by the Ethics Committee of the Second Xiangya Hospital, Central South University.

### 2.5. Extraction of mice bone marrow cells

After being sacrificed, the mice’s tibias were isolated. The epiphyseal was cut off, and the bone marrow fluid was rinsed with normal saline. After filtering with a 70 μm cell screen, the single-cell suspension was prepared.

### 2.6. Extraction of mice peritoneal macrophages

After being sacrificed, the mice were intraperitoneally injected with 5 ml RPMI-1640, and the abdomen was gently rubbed for 2–3 min. The syringe was pumped back to extract a suspension rich in macrophages. The extracted cells were resuspended in complete medium and cultured in a 5% CO_2_ incubator at 37°C for 2 hours. The supernatants were discarded and rinsed 3 times with PBS, and the adherent cells were monolayers of macrophages. After adding an appropriate amount of trypsin for digestion, the medium was added to the complete medium and pipetted into a single cell suspension by pipette, then washed 3 times with PBS and collected.

### 2.7. Extraction of mice spleen lymphocytes

After removal, the spleen was gently ground on a 70 μm cell sieve to prepare a single-cell suspension. Then, the cell suspension was carefully and slowly added to the liquid surface of the separation solution. After centrifugating at 2500 r/min for 25 min, the second layer of cloud-like lymphocyte layer was aspirated and resuspended in PBS. After centrifugating at 1500 r/min for 5 min, the spleen lymphocytes were collected.

### 2.8. Neutralization of TSLP

Anti-TSLP mAb (Biolegend, #512206) was added to the culture system (20 μg/ml, 24 h) in vitro experiment.

### 2.9. Flow cytometry analysis

After the cleavage of red blood cells by Red Blood Cell Lysis Buffer (BD Pharmingen, San Jose, CA, USA), lymphocyte suspensions were obtained and washed in PBS. Then the cells were fixed, permeabilized, and stained at 4°C with antibodies before analysis. Antibodies used are listed in Table S1.

### 2.10. Quantitative real-time PCR (qRT-PCR).

qRT-PCR was performed as previously described.^6^ The expression of mRNAs was normalized to GAPDH, while the expression of miRNAs was normalized to U6. Gene expression were calculated by the 2^−ΔΔCt^ method.^22^ Primers were listed in Table S2.

### 2.11. Western blot

Proteins were harvested using RIPA lysis buffer (Beyotime, #P0013B), then separated with 10% SDS-PAGE, transferred onto PVDF membrane, and blocked with 5% skim milk powder at room temperature for 2 h. The PVDF membranes were incubated with primary antibodies (listed in Table S1) at 4°C overnight and then incubated with HRP-labeled secondary antibodies. ECL System (Azure Biosystems, Dublin, CA, USA) was used to develop the films.

### 2.12. Cell transfection

siRNAs were purchased from Ribobio Biotech (Guangzhou, China). The siRNAs and negative controls were transfected into cells using Lipofectamine 2000 (Invitrogen, #11668030) for 72 h. The siRNAs sequences: si-h-IDO1 GAAAGAGTTGAGAAGTTAA; si-h-IDO2 GGAGCTACCATCTGCAAAT; si-h-IDO3 GAACGGGACACTTTGCTAA.

The inhibitor and mimics of miR-27a-3p were purchased from GenePharma (Suzhou, China). M2 macrophages were constructed as described above and then transfected using TransIT-X2 (Mirusbio, #MIR 6003) for 72 h. Inhibitor sequence: S: GCGGAACUUAGCCACUGUGAA; Mimics sequence: S: UUCACAGUGGCUAAGUUCCGC, AS: GGAACUUAGCCACUGUGAAUU.

### 2.13. Enzyme-linked immunosorbent assay (Elisa) for cytokines

The levels of IL-4 (Elabscience, #E-EL-H0101c), IL-5 (Elabscience, #E-EL-H0191c), IL-13 (Elabscience, #E-EL-H0104c), and TSLP (Elabscience, #E-EL-H1598c; Jonln, #JL20618-96T) in the cell supernatants were measured by the ELISA kit following the manufacturer’s instructions. Concentration was calculated according to the standard curve.

### 2.14. Statistical analysis

Each experiment was repeated at least 3 times. Data were analyzed with GraphPad Prism 8.0. Data normality was confirmed by the Shapiro – Wilk normality test and data were presented as the Means ± standard deviations. Student’s t-test was used to analyze the difference between two groups. For multiple comparisons, one-way ANOVA was performed, followed by Dunnett correction. Correlations were analyzed using Spearman’s correlation. *p* < .05 was considered as statistical significance.

## 3. Results

### 3.1. ILC2 activation caused by H. pylori infection requires macrophages

The proliferation and activation of ILC2 are mainly affected by the transcription factor GATA-3 in bone marrow, stimulation of mucosal barrier by pathogens, and interaction with other immune cells.^23,24^ Therefore, we first detected the ILC2 and GATA-3^+^ cells ratio in the bone marrow of mice infected with *H. pylori* for 12 weeks and 24 weeks, respectively. Data showed no significant differences between the *H. pylori* infection groups and the negative control group (Figure S1). Therefore, the activation of ILC2 in *H. pylori* infection is not exerted by affecting GATA-3 in the bone marrow.

**Figure 1.**
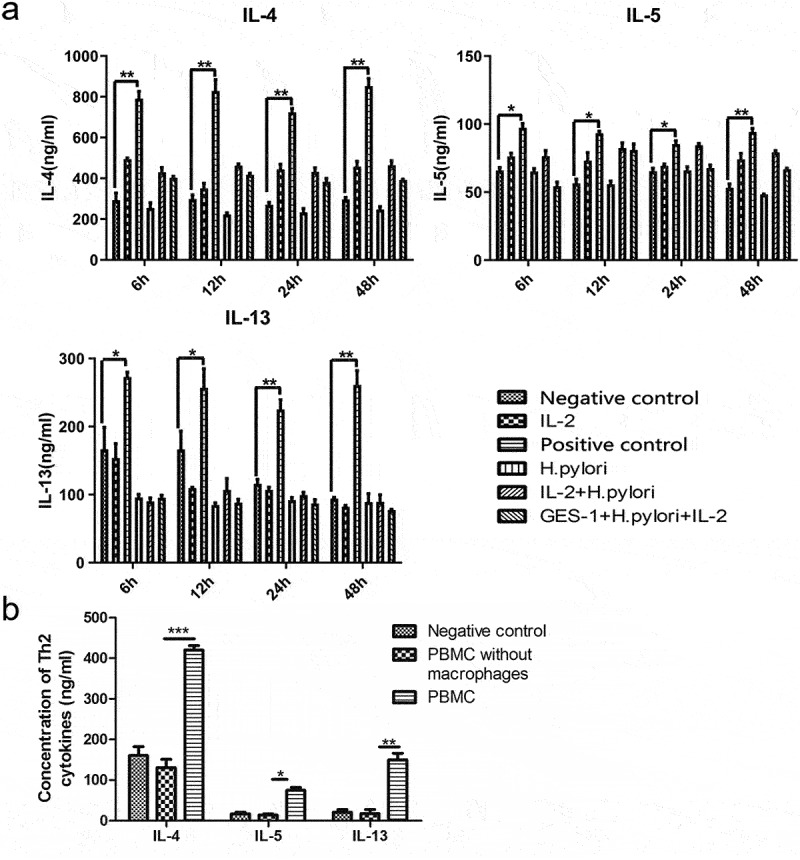
Macrophages were required in the activation of ILC2 induced by *H. pylori* infection. (a) ILC2 sorted out from PBMC were cocultured with *H. pylori* or together with GES-1. The IL-2 +IL-25 +IL-33 stimulating group was used as positive control. Elisa showed the level of Th2 cytokines (IL-4, IL-5, and IL-13) in the supernatants; (b) PBMC removing or remaining macrophages were cocultured with GES-1 and *H. pylori* ATCC43504 for 24 hours. The Th2 cytokines in the supernatants were detected by Elisa. *p* values were determined by one-way ANOVA, Dunnett-adjusted; error bars, SD (*n* = 3 per group); *p < .05, **p < .01, ***p < .001.

Stimulation of the mucosal barrier by pathogens is another critical factor facilitating the proliferation and activation of ILC2.^24^ When infection occurs, the mucosal barrier releases “alarmin” proteins such as IL-25, IL-33, and TSLP, which can activate ILC2 and help to trigger the type-2 immunity process.^25^ In a previous study, we proved that *H. pylori* infection promoted ILC2 in the gastric mucosa or PBMC. Here, we further sorted out ILC2 from the PMBC and cocultured them with *H. pylori* or together with GES-1. Elisa results showed that the level of Th2 cytokines in the supernatants did not increase when stimulating ILC2 with *H. pylori* or *H. pylori*-infected GES-1 cells, regardless of different time points or different MOI (Figure 1a, Data only showed the MOI of 50). These observations revealed that *H. pylori* did not activate ILC2 directly and some other immune cells may serve as intermediaries. As classic participants in innate immunity, macrophages also respond immediately at the early stage of disease. Interestingly, in the coculture system (*H. pylori*, GES-1, and PBMC), the Th2 cytokines of the supernatants increased. When macrophages were removed, no significant changes were observed (Figure 1b). These findings suggested that ILC2 activation caused by *H. pylori* infection requires macrophages.

### 3.2. H. pylori infection promoted macrophages M2 polarization to activate ILC2

**Figure 2.**
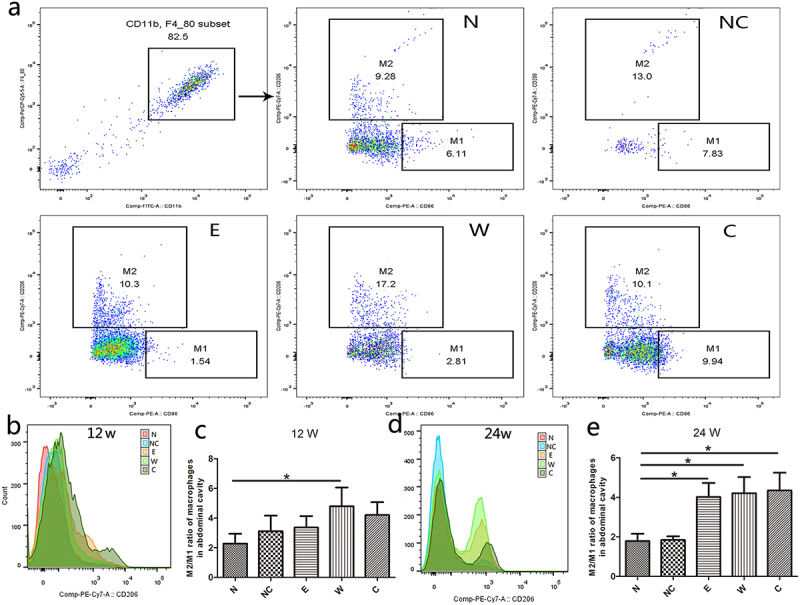
*H. pylori* infection promotes M2 polarization in peritoneal macrophages. *H. pylori*-infected mice were sacrificed at the 12th week or 24th week. (a) Flow cytometry was used to detect the peritoneal macrophages, and the extraction efficiency of mice peritoneal macrophages was more than 80%. (b, d) the expression of CD206 in mice peritoneal macrophages. (c, e) the ratio of M2/M1 macrophages. N: mice group with no treatment, NC: mice group treated with PBS, E: mice group treated with *H. pylori* strain of East Asian type, W: mice group treated with *H. pylori* strain of Western type *SS1*, C: mice group treated with CagA- *H. pylori*. *p* values were determined by one-way ANOVA, dunnett-adjusted; error bars, SD (*n* = 6 per group); *p < .05.

**Figure 3.**
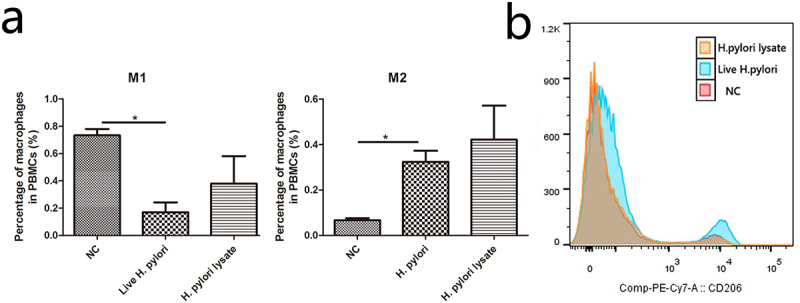
*H. pylori* infection promotes M2 polarization of macrophages in PBMC. In the *H. pylori* or the lysate of *H. pylori* coculture system: (a)The percentages of M1 and M2 macrophages were analyzed with flow cytometry. (b) The expression of CD206 in PBMC. NC: negative control. *p* values were determined by one-way ANOVA, Dunnett-adjusted; error bars, SD (*n* = 3 per group); *p < .05.

To investigate how macrophages help to activate ILC2, we extracted mice peritoneal macrophages when mice were infected with *H. pylori* for 12 weeks and 24 weeks. Compared with the negative control group, the M2/M1 ratio of macrophages was increased in *H. pylori* infection groups, especially in the 24-week groups (Figure 2). Although, the total number of peritoneal macrophages among groups showed no significant differences (Figure S2). Meanwhile, GES-1 stimulating with live *H. pylori* or *H. pylori* lysate was cocultured with human PBMC. Data showed that the percentage of M1 macrophages was reduced while the percentage of M2 macrophages was augmented in the live *H. pylori* group but not the *H. pylori* lysate group (Figure 3a). Consistently, the expression of CD206, a M2 macrophage marker, was up-regulated in PBMC when stimulated with live *H. pylori* (Figure 3b). These results demonstrated that *H. pylori* infection promoted macrophages M2 polarization.

Since M2 macrophages have been proven to promote and cooperate with ILC2 in type-2 immunity,^16,17^ we analyzed the percentages of ILC2 and M2 macrophages in the coculture system (*H. pylori*, GES-1, and PBMC), and a positive correlation was found between the two (R^2^ = 0.914, *p*=.011, Figure S3a). As for *H. pylori*-infected mice, the percentage of ILC2 in spleen lymphocytes and the percentage of M2 macrophages in peritoneal cavity were positively corrected too (R^2^ = 0.6835, *p* < .001, Figure S3b). These results indicated that *H. pylori* infection activated ILC2 by promoting macrophages M2 polarization.

### 3.3. Indoleamine 2,3-dioxygenase (IDO) and TSLP were involved in the “H. pylori infection- M2 polarization- ILC2 activation” pathological process

**Figure 4.**
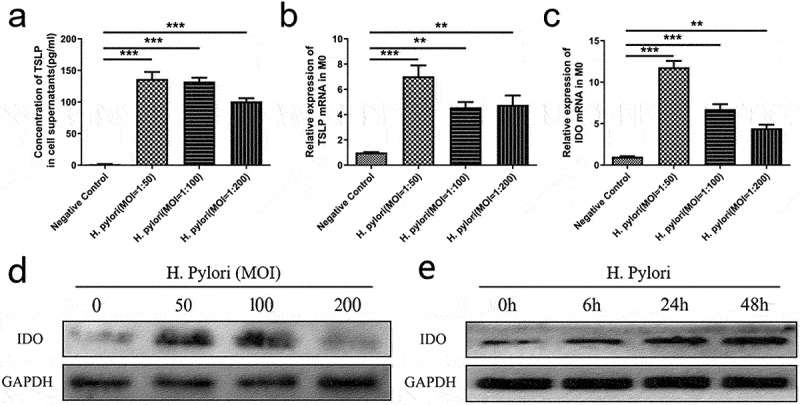
*H. pylori* infection promotes the expression of IDO and TSLP in macrophages. M0 macrophages were infected with *H. pylori* ATCC43504. (a) The concentration of TSLP in the supernatants. (b, c) the mRNA expression of TSLP and IDO. (d) The expression of IDO protein at different MOI. (e) The expression of IDO protein at different time points (MOI = 50). MOI: multiplicity of infection. *p* values were determined by one-way ANOVA, Dunnett-adjusted; error bars, SD (*n* = 3 per group); **p < .01, ***p < .001.

Next, we explored the mechanism by which *H. pylori* infection induced M2 polarization. We conducted gene expression profiling of M0 macrophages, which were stimulated by *H. pylori* for 24 hours. A total of 22 genes were significantly up-regulated and 16 genes were significantly down-regulated (Figure S4 &amp; Table S3). Among them, IDO and TSLP aroused our interest. IDO has been known to induce macrophages M2 polarization, and TSLP is one of the well-known classical “alarmin” to activate ILC2. Further experiments showed that after *H. pylori* infection, the concentration of TSLP in cell supernatants and the mRNA expression of TSLP in M0 macrophages were enriched (Figure 4a, b). The expression of IDO was also up-regulated both in mRNA and protein levels (Figure 4c–e). Undoubtedly, IDO and TSLP were involved in the “*H. pylori* infection- M2 polarization- ILC2 activation” pathological process.

**Figure 5.**
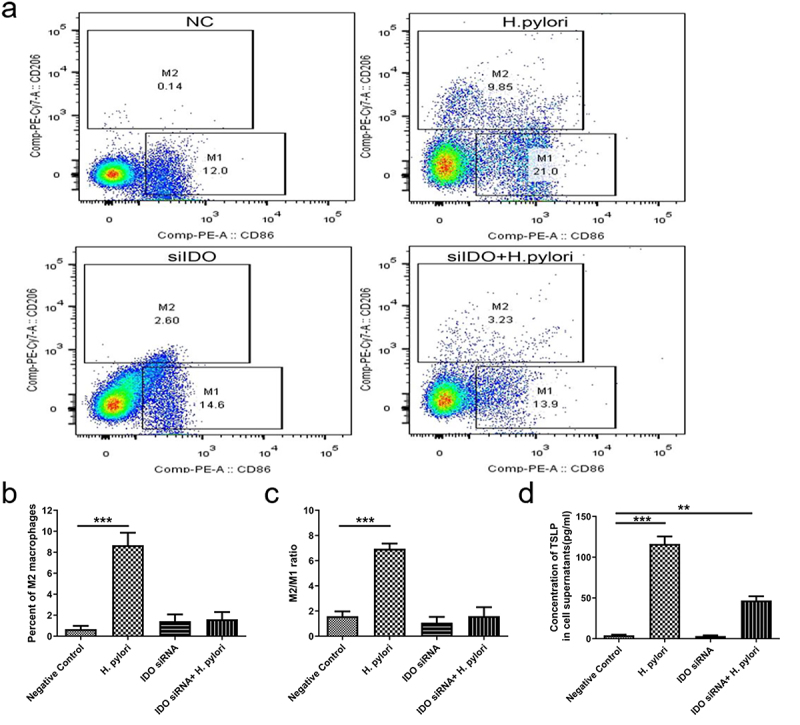
The expression of IDO is vital for macrophage M2 polarization in *H. pylori* infection. M0 macrophages were transfected with IDO siRnas and cocultured with *H. pylori* ATCC43504 for 24 hours. (a-c) flow cytometry was used to analyze the proportion of M2 macrophages (a, b) and the ratio of M2/M1 macrophages (c). (d) The concentration of TSLP in the supernatants. NC: negative control. *p* values were determined by one-way ANOVA, Dunnett-adjusted; error bars, SD (*n* = 3 per group); **p < .01, ***p < .001.

**Figure 6.**
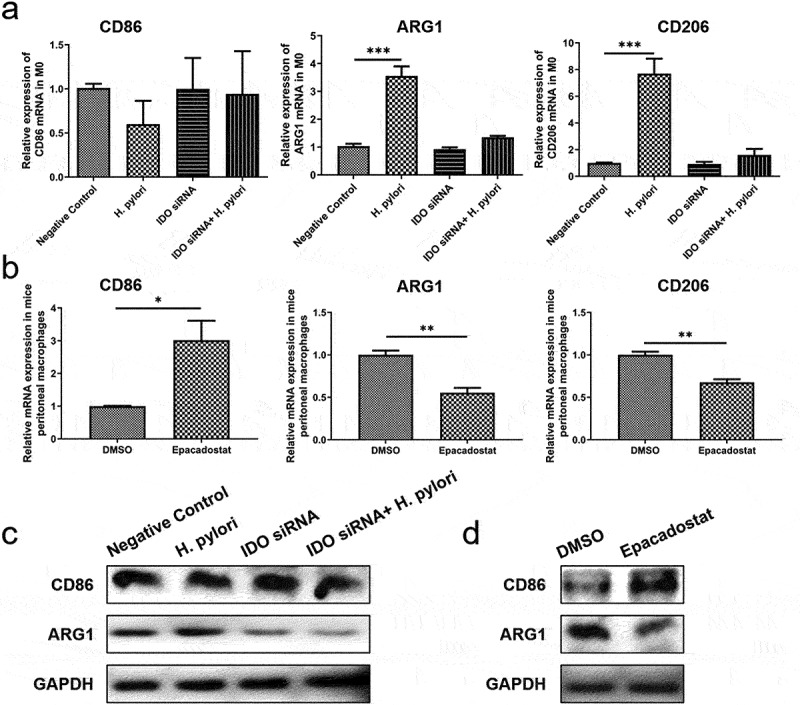
The influence of IDO on the expression of M1/M2 markers. (a, b) the mRNA expression of M1/M2 markers in M0 macrophages (a) and mice peritoneal macrophages (b). (c, d) the protein expression of M1/M2 markers in M0 macrophages (c) and mice peritoneal macrophages (d). *p* values were determined by one-way ANOVA followed by Dunnett correction (a) or Student’s t-test (c); error bars, SD (*n* = 3 per group); *p < .05, **p < .01, ***p < .001.

Next, we knocked down IDO in M0 macrophages with specific siRNA and then cocultured them with *H. pylori* for 24 hours. Flow cytometric analysis showed that *H. pylori* infection increased the proportion of M2 macrophages and the concentration of TSLP, while the transfer of siIDO counteracted this effect (Figure 5). The mRNA and protein expression of M1 molecular marker CD86 was not affected by *H. pylori* or siIDO. In contrast, the promotion effect of *H. pylori* on M2 markers ARG1 and CD206 was weakened by siIDO (Figures 6a & 6c). Then, the peritoneal macrophages from *H. pylori SS1* infected mice (12 weeks after infection) were treated with Epacadostat (IDO inhibitor, 50 nM for 48 h). We found that IDO inhibiting significantly up-regulated M1 marker and down-regulated M2 markers (Figures 6b & 6d). These results declared that IDO was required for the induction and maintenance of macrophage M2 polarization in *H. pylori* infection.

### 3.4. TSLP played a critical role in the activation of ILC2 in H. pylori infection

**Figure 7.**
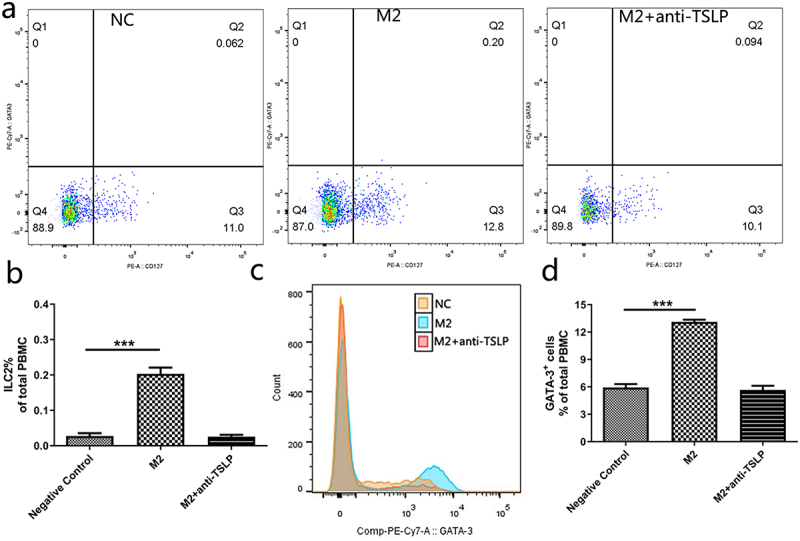
TSLP secreted by M2 macrophages increases the proportion of ILC2 in PBMC. (a) Three groups were cocultured with *H. pylori*: negative control group (NC), M2 macrophage group (M2), and anti-TSLP group (M2+anti-TSLP). (b) The proportion of ILC2 in PBMC. (c) The count of GATA-3^+^ cells in PBMC. (d) The proportion of GATA-3^+^ cells in PBMC. NC: negative control. *p* values were determined by one-way ANOVA, Dunnett-adjusted; error bars, SD (*n* = 3 per group); ***p < .001.

As described above, TSLP might be the central member to mediate ILC2 activation in *H. pylori* infection. In order to verify this, M2 macrophages were treated with anti-TSLP and then cocultured with PBMC. We found M2 macrophages boosted the proportion of ILC2 and GATA-3^+^ cells in PBMC. However, anti-TSLP treatment abolished this effect completely (Figure 7), which proved that TSLP was the key player in M2 macrophages-mediated ILC2 activation.

Hence, we intended to dig out how *H. pylori* infection up-regulated the expression of TSLP in macrophages. Since microRNAs are potent genetic regulators, miR-27a-3p and miR-1956 came into view. They were the top two down-regulated miRNAs in the gene expression profiling of *H. pylori*-infected M0 macrophages (Figure S4 &amp; Table S3). Target prediction using Targetscan (http://www.targetscan.org/) indicated that TSLP could be targeted by miR-27a-3p (Figure S5a). Then, we infected M0 macrophages with *H. pylori* in different MOI and found that miR-27a-3p was negatively correlated with the expression of TSLP (Figure S5b). We consequently transfected M2 macrophages with miR-27a-3p inhibitors or mimics. Anticipatedly, knocking down miR-27a-3p raised the mRNA expression and the protein secretion of TSLP, while transfection of mimics curbed it (Figure S5c-e). Based on this, we proposed that *H. pylori* infection inhibited the expression of miR-27a-3p in macrophages, thus boosting the production and secretion of its target protein TSLP and then promoting the activation of ILC2.

## 4. Discussion

**Figure 8.**
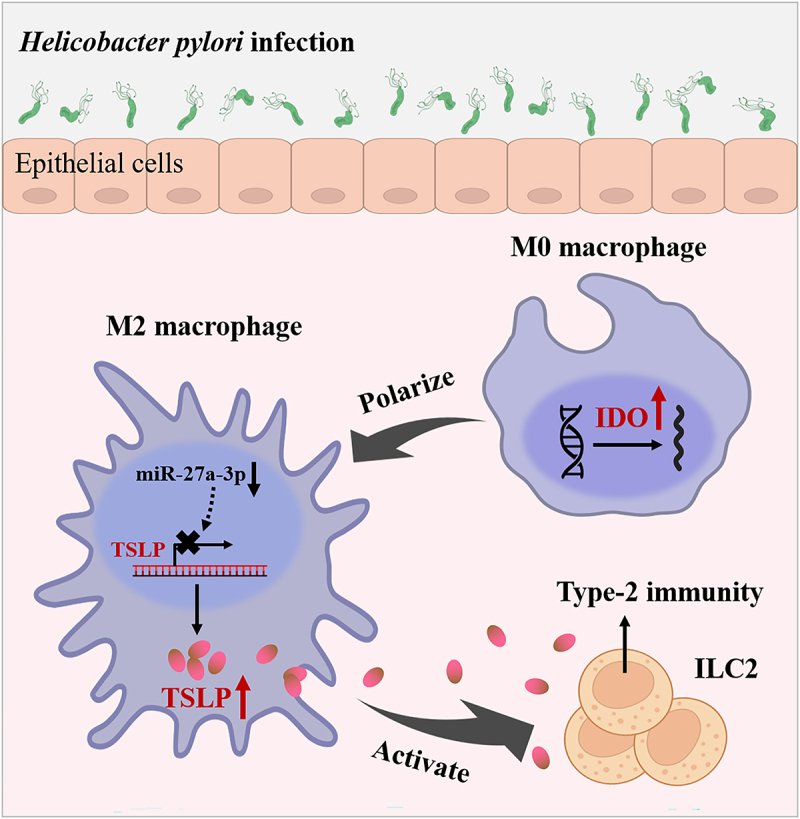
A schematic chart of *H. pylori* infection-induced ILC2 activation mechanism.

Our previous study demonstrated that the activation of ILC2 drove skewed type-2 immunity in *H. pylori* infection-associated gastric disease.^6^ In this research, we described a novel mechanism involving macrophage M2 polarization as an essential procedure promoting ILC2 activating in *H. pylori* infection. In addition to that, IDO and TSLP were identified as the fat parts of this process (Figure 8).

The role of ILC2 in *H. pylori* infection has come into focus over the past few years. Stomach ILC2 proliferation and IL-5 secretion provoked IgA-mediated *H. pylori* elimination and gastric inflammation restraint in the early phase of *H. pylori* infection.^26^ We have also observed the proliferation of ILC2 as well as the activation of the type-2 immunity in gastric tissue. ILC2 and its mediated type-2 immunity play a vital role in mucosal healing and homeostasis. Additionally, some human populations with equipollent *H. pylori* infection rates but a high gut parasite burden have a lower incidence of gastric cancer.^27^ Thus, we share views with some researchers that mechanisms promoting type-2 immunity can limit *H. pylori*-induced gastric pathology.^28^

It is reported that the number of macrophages in the gastric mucosa of *H. pylori* infection is significantly increased.^29^ Moreover, some studies also showed that macrophages extracted from the peritoneal cavity of *H. pylori*-infected mice or *H. pylori*-stimulated macrophages in vitro may play a role in regulating the transformation of Th1/Th2 immune balance.^30,31^ In our system, we removed the macrophages from PBMC and cocultured the remaining cells with *H. pylori* in vitro. As anticipated, ILC2 could not be activated after the removal of macrophages, which suggests the indispensable role of macrophages.

Macrophages can be polarized to M1 or M2 in different microenvironments. Studies have found that the molecular marker CD163 of M2 macrophages was significantly increased in *H. pylori* ^+^ gastric cancer tissues compared with that in *H. pylori* ^−^ tissues.^32,33^ Our findings indicated that *H. pylori* infection could promote the polarization of macrophages to M2. We also confirmed that the proportion of ILC2 was positively correlated with the proportion of M2 macrophages after *H. pylori* infection. This is in line with a recent report that M2 macrophages promoted ILC2 expansion and mucous metaplasia in early-life rhinovirus infections.^17^ These observations, together with our findings presented here, indicate that M2 polarization of macrophages is involved in the activation of ILC2 induced by *H. pylori* infection.

One thing to note here is that in our model, *H. pylori* infection conducted to M2 phenotype in a CagA-independent manner (Figure 2). Our finding is in keeping with a previous study that the mRNA expression of the M2 marker CD163 was higher in *H. pylori*-infected relative to noninfected patients, while no significant difference was found in *H. pylori* CagA^+^ relative to *H. pylori* CagA^−^ patients.^29^

Multiple signals are involved in the M2 polarization of macrophages.^34^ Herein, we demonstrated for the first time that IDO, the rate-limiting enzyme of tryptophan catabolism, contributed to the *H. pylori* infection-induced M2 polarization. Various pathogens such as viruses,^35,36^ bacteria,^37,38^ fungi,^39,40^ and parasites^41^ could activate IDO in macrophages and modulate inflammation. IDO-expressing macrophages were deemed to skew differentiation toward the M2 phenotype.^37^ Our results are consistent with previous studies that elevated IDO attributed to the predominance of M2-like phenotype in lepromatous leprosy^42^ and *Schistosoma japonicum*^41^ infection. However, some studies reported that *Fusobacterium nucleatum*^43^ and *Aspergillus fumigatus*^39^ infection induced IDO expression and M1 polarization. Such differences may result from the species and virulence of pathogens; the conditions, MOI, and course of infection. Anyhow, IDO is critical for regulatory macrophage function and mediating inflammatory response in pathogen infection.

Furthermore, we found that TSLP secreted by macrophages is requisite to promote ILC2 as anti-TSLP neutralized this effect. Like IL-25 and IL-33, TSLP is a classic “alarmin” to activate ILC2.^44^ We also found out that miR-27a-3p is a potential regulator of TSLP, since transfection of inhibitors or mimics influenced the secretion of TSLP in M2 macrophages.

In conclusion, our study highlights that M2 macrophages contribute to the activation of ILC2 in *H. pylori* infection. We demonstrate that *H. pylori* infection can induce high expression of IDO and M2 polarization of macrophages, which subsequently secret TSLP and finally activate ILC2 (Figure 8).

## Supplementary Material

Supplemental Material

## Data Availability

The authors confirm that the data supporting the findings of this study are available within the article and its supplementary materials.
