# Supplementary material for: M2 macrophages participate in ILC2 activation induced by Helicobacter pylori infection
Source: Gut Microbes. 2024 May 1;16(1):2347025. doi: 10.1080/19490976.2024.2347025 (PMC11067991; doi:10.1080/19490976.2024.2347025)
Supplement: Supplemental Material [file KGMI_A_2347025_SM1712.zip › Supplementary figures (1).doc]

Supplementary figures

Figure S1

Figure S1. *H. Pylori* infection does not affect the ratio of ILC2 and GATA-3^+^ cells in mice bone marrow. Mice infected with *H. Pylori* were sacrificed at the 12th week (a) or 24th week(b), and bone marrow cells were extracted from tibias. Flow cytometric analysis showed no significant change in the ratio of ILC2 and GATA-3^+^ cells between each group. N: mice group with no treatment, NC: mice group treated with PBS, E: mice group treated with *H. Pylori* strain of East Asian type, W: mice group treated with *H. Pylori* strain of Western type *SS1*, C: mice group treated with CagA^-^ *H. Pylori*. The data were expressed as means ± SD (n=6 per group), and significance was tested using the Dunnett test.

Figure S2

Figure S2. *H. Pylori* infection does not affect the total number of mice peritoneal macrophages. Peritoneal macrophages were isolated from *H. Pylori*-infected mice. Flow cytometry showed no differences in the number of peritoneal macrophages among groups at the 12th week (a) or 24th week (b). N: mice group with no treatment, NC: mice group treated with PBS, E: mice group treated with *H. Pylori* strain of East Asian type, W: mice group treated with *H. Pylori* strain of Western type *SS1*, C: mice group treated with CagA^-^ *H. Pylori*. The data were expressed as means ± SD (n=6 per group), and significance was tested using the Dunnett test.

Figure S3

Figure S3. Correlation analysis between the proportion of ILC2 cells and the proportion of M2 macrophages after *H. Pylori* infection. (a) In the coculture system, there was a positive correlation between the ratio of ILC2 cells and M2 in PBMC after *H. Pylori* ATCC43504 stimulation. (b) In *H. Pylori*-infected mice (*H. Pylori* strains of East Asian type, Western type *SS1*, and CagA^-^ type), the proportion of spleen ILC2 cells was positively correlated with the proportion of M2 in the abdominal cavity.

Figure S4

Figure S4. Analysis of gene expression profiling results. (a, b) After *H. Pylori* ATCC43504 stimulating (MOI=1: 50) for 24h, 22 genes were up-regulated, and 16 genes were significantly down-regulated in M0 macrophages. (c) The expression changes of TSLP and IDO in macrophages after *H. Pylori* ATCC43504 stimulation.

Figure S5

Figure S5. TSLP could be the target of miR-27a-3p. (a) The TSLP gene might be a target for miR-27a-3p predicted by TargetScan (<http://www.targetscan.org/>). (b) The expression of TSLP and miR-27a-3p were negatively correlated when treating M0 macrophages with *H. pylori* ATCC43504 in different MOI for 24h. (c) The miR-27a-3p and TSLP expression of M2 macrophages transfected with miR-27a-3p inhibitors. (d) The miR-27a-3p and TSLP expression of M2 macrophages transfected with miR-27a-3p mimics. (e) The concentration of TSLP in cell supernatants of M2 macrophages transfected with miR-27a-3p inhibitors or mimics. *P* values were determined by Student’s t-test; Error bars, SD (n=3 per group); ***P*<0.01, ****P*<0.001.
